# Sustained yet non-curative response to lenalidomide in relapsed angioimmunoblastic T-cell lymphoma with acquired chidamide resistance: a case report with 10-year follow-up, genetic insights and literature review

**DOI:** 10.3389/fonc.2024.1471090

**Published:** 2024-11-27

**Authors:** Juan Xu, Jie Huang, Liping Xie, Ting Liu, Jianjun Li, Xinchuan Chen, Zhigang Liu, Sha Zhao, Caigang Xu, Yu Wu

**Affiliations:** ^1^ Department of Hematology, Institute of Hematology, West China Hospital, Sichuan University, Chengdu, China; ^2^ Department of Pathology, West China Hospital, Sichuan University, Chengdu, China; ^3^ Chengdu Shang Jin Nan Fu Hospital / Shang Jin Hospital of West China Hospital, Sichuan University, Chengdu, China

**Keywords:** angioimmunoblastic T-cell lymphoma, relapsed, lenalidomide, chidamide, resistance, immune dysregulation

## Abstract

Angioimmunoblastic T-cell lymphoma (AITL) is an aggressive subtype of peripheral T-cell lymphoma (PTCL) characterized by its T-follicular helper (TFH) phenotype. Relapsed and refractory disease is common in AITL and often associated with a poor prognosis. The presence of epigenetic abnormalities, immune dysregulation, hyperinflammation and active angiogenesis in AITL offers potential targets for histone deacetylase (HDAC) inhibitors and immunomodulatory drugs (IMiDs). Herein, we present a case of AITL with multiple relapses over a decade. Following intensive chemotherapy and autologous stem cell transplantation (ASCT), the patient relapsed with extensive nodal and extranodal involvement, particularly pulmonary lesions, and subsequently pursued chemo-free treatments. Initially, the patient exhibited a remarkable response to single-agent chidamide, the first oral HDAC inhibitor. Soon after developing resistance to chidamide, continuous treatment with lenalidomide led to an impressive sustained complete remission lasting 64 months, followed by a diminished response for an additional 11 months. Genetic profiling of the patient revealed mutations in KMT2D and ARID1A, along with chromosomal aberrations such as del(5q). Notably, genes commonly mutated in AITL, including RHOA, TET2, DNMT3A, and IDH2, were absent in this case. A review of the literature highlights the heterogeneous genomic landscape of AITL and the diversity of treatment options available, underscoring the importance of tailored approaches to overcome resistance and improve outcomes in this distinct lymphoma subtype.

## Introduction

Angioimmunoblastic T-cell lymphoma (AITL), recently classified under nodal T-follicular helper lymphomas (nTFHLs) (1), represents a significant subtype of peripheral T-cell lymphoma (PTCL), accounting for approximately 1-2% of all non-Hodgkin lymphomas (2). AITL is characterized by a minority population of neoplastic T-follicular helper (TFH) cells residing in a complex tumor microenvironment rich in follicular dendritic cells (FDC), high endothelial venules (HEV), and polymorphic inflammatory cells (3, 4). The normal TFH counterparts are crucial for germinal center formation and activation, aiding in the maturation and differentiation of B cells into plasma cells and memory B cells (5). Consequently, AITL typically presents with immune dysregulation, manifesting as hypergammaglobulinemia, autoimmune phenomena, opportunistic infections, prevalent Epstein-Barr virus (EBV) infection, and sometimes featuring prominent or even clonal expansion of bystander B cells or plasma cells (2, 3, 5–11). Additionally, AITL exhibits a unique genetic profile, frequently harboring specific driver mutations like IDH2^R172K^ and RHOA^G17V^, as well as mutations in epigenetic regulatory genes, such as TET2 and DNMT3A, and T-cell receptor (TCR)-related genes (1, 5, 12).

AITL is often associated with advanced-stage disease, an aggressive clinical course, and generally unfavorable prognosis (2). The anthracycline-based regimens with or without etoposide have been widely adopted as first-line treatment options, demonstrating a 5-year overall survival (OS) rate of 32-44% (2, 13–17). Upfront consolidation with high-dose chemotherapy and autologous stem cell transplantation (HDC/ASCT) has shown a survival advantage in younger patients with chemosensitive disease (16). However, relapsed or refractory disease after ASCT is common, and these patients typically experience poor outcomes (18). The optimal salvage therapy has yet to be established, with conventional chemotherapy usually providing only palliative benefits (15). Therefore, based on an improved understanding of the molecular pathogenesis of AITL, various targeted agents, such as epigenetic regulators and immunomodulators, have demonstrated potential in managing relapsed or refractory cases (19). Nonetheless, their appropriate maintenance treatment course and curative efficacy remain undetermined.

Here, we present a case of AITL that relapsed with extensive nodal and extranodal involvement, primarily in the lungs, following multiple lines of chemotherapy and ASCT. Notably, the patient exhibited a remarkable response to single-agent chidamide, and even more impressively, followed by a prolonged remission with lenalidomide after developing resistance to chidamide. Despite the promising outcome of this chemo-free salvage treatment approach, the patient ultimately experienced disease relapse and was unable to achieve a cure for this lymphoma. To gain further insight, his genetic profile was analyzed throughout the disease course.

## Case presentation

A 54-year-old Chinese man presented to a local hospital in October 2013 with a two-month history of worsening dyspnea and dry cough, accompanied by pruritic rash, night sweats and weight loss. He had no significant comorbidities. Examination revealed generalized lymphadenopathy and marked hypereosinophilia (eosinophils: 10.86×10^9/L in the peripherial blood, accounting for 72.1% of white blood cells, and 37.5% of all nucleated cells in the bone marrow). No rearrangements of PDGFRA, PDGFRB or FGFR1 were detected by fluorescence *in situ* hybridization (FISH) analysis. AITL was diagnosed through a cervical lymph node biopsy and subsequent pathological consultation at our hospital, revealing typical features: dilated sinuses, follicle depletion, prominent arborizing HEV, polymorphic cellular infiltration, an expanded FDC network, and neoplastic T cells with clear cytoplasm partially expressing CXCL13 and PD-1 (**Supplementary Figure S1**). Clonal T-cell receptor (TCR) rearrangement was also detected. Notably, EBV-positive cells were absent in this case. The patient was diagnosed with stage IVB disease, with extensive nodal and pulmonary involvement shown on positron emission tomography/computed tomography (PET/CT) scan. A complete response (CR) was achieved with the CHOP (cyclophosphamide, doxorubicin, vincristine, and prednisone) regimen. However, regrowth of a lymph node in the right axilla occurred after the fifth cycle of chemotherapy. The patient was then referred to our institution in February 2014, where an excisional biopsy confirmed the relapse of AITL. PET/CT imaging revealed increased uptake in the maxilla, nasal root, and right shoulder. Additionally, bone marrow examination continued to show elevated eosinophils without evidence of lymphoma infiltration. After two cycles of second-line chemotherapy with gemcitabine and oxaliplatin (GemOx), PET/CT was completely negative. Subsequently, the patient underwent a third cycle followed by consolidation with BEAM (carmustine, etoposide, cytarabine, and melphalan)-conditioned ASCT in June 2014. Interleukin-2 was administered as maintenance therapy thereafter.

**Figure 1:**
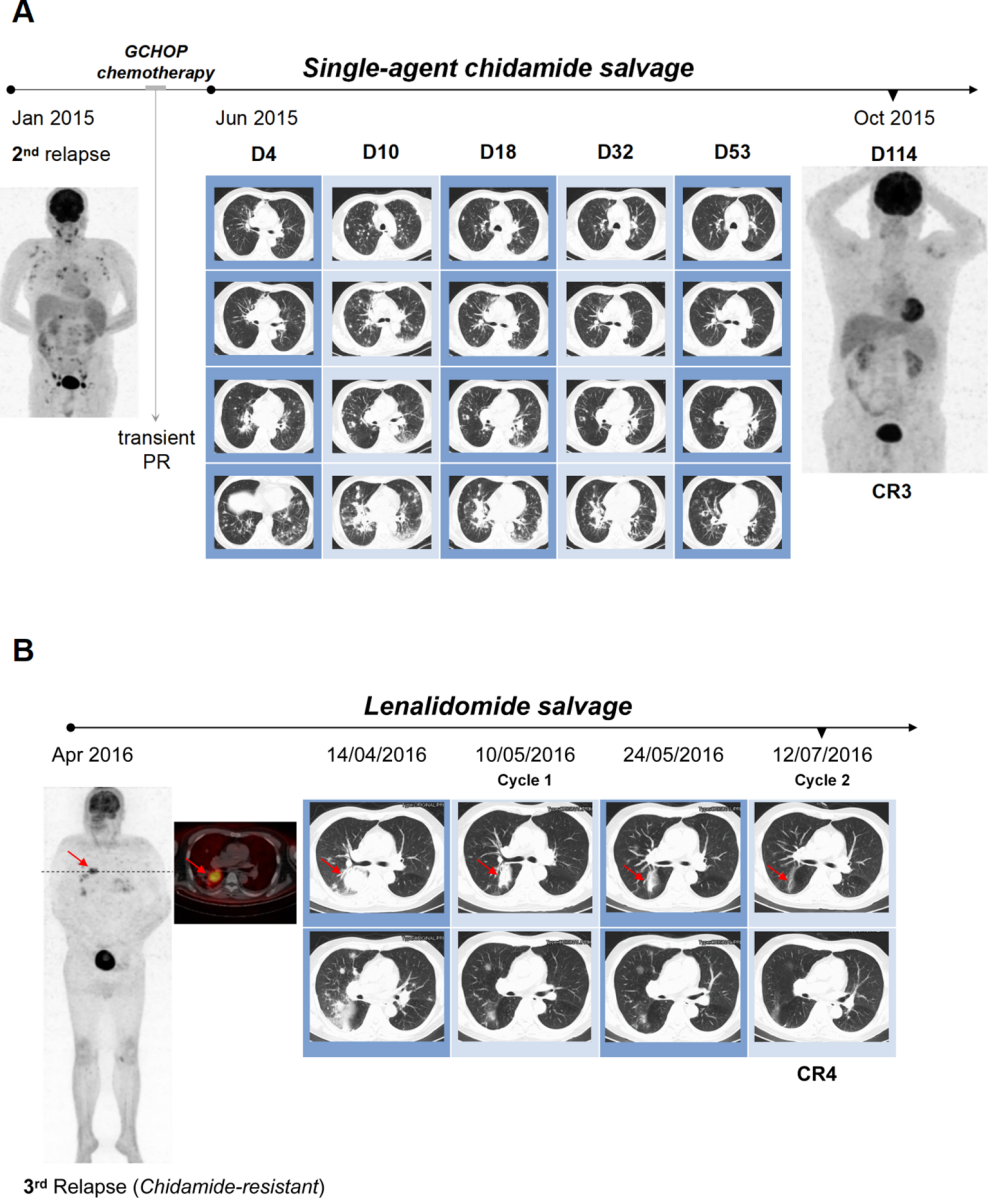
Achieving dramatic response in pulmonary lesions with chemo-free salvage. **(A)** The patient experienced second AITL relapse with widespread lymphadenopathy in Jan 2015. GCHOP chemotherapy resulted in transient PR, but disease progressed in May 2015 with multiple bilateral pulmonary nodules and masses on chest CT. Salvage therapy with single-agent chidamide was initiated in June 2015. Despite initial lesion size increase within the first 10 days, pulmonary lesions gradually decreased on subsequent CT scans, achieving significant diminution by week 8 (day 53) and a third CR by week 16 (day 114). **(B)** Third CR achieved with chidamide was maintained until April 2016, when the patient relapsed again with notable bilateral pulmonary masses. Salvage therapy started with lenalidomide and prednisone. Prednisone was tapered off over a month, after which therapy switched to single-agent lenalidomide. Follow-up radiographs showed remarkable resolution of all pulmonary lesions after two cycles, resulting in a fourth CR in July 2016.

In January 2015, 7 months post-transplantation, the patient presented with high fever, peripheral edema and progressive lymphadenopathy, with no eosinophilia detected. PET/CT imaging indicated uptake in the cervical, supraclavicular, axillary, mediastinal, hilar, abdominal, pelvic and groin nodes (**Figure 1A**). A second relapse of AITL was diagnosed following an inguinal lymph node biopsy. Moreover, the infiltrating lymphoma cells were negative for Epstein-Barr virus-encoded small RNA (EBER) by *in situ* hybridization. Three cycles of GCHOP (gemcitabine, cyclophosphamide, epirubicin, vindesine and dexamethasone) only resulted in a transient partial remission (PR). A chest CT scan in May 2015 revealed multiple bilateral pulmonary nodules and masses. These lesions showed some response to corticosteroids yet remained unresponsive to anti-infective medications, indicating disease progression. Despite recommendations for a biopsy procedure, consent was declined. The patient’s condition deteriorated rapidly. In June 2015, single-agent chidamide was administered orally at a dosage of 30mg twice per week as salvage therapy. Following an initial enlargement, the pulmonary lesions exhibited a gradual reduction, ultimately reaching a noteworthy diminishment by week 8 and a third CR by week 16 (**Figure 1A**).

Despite experiencing grade 2 cytopenia, fatigue, and diarrhea, the patient continued chidamide treatment, and remission was sustained until April 2016. At that time, the patient developed dyspnea, though no palpable peripheral lymphadenopathy was present. However, a chest CT demonstrated the recurrence of sizable pulmonary masses, and PET/CT imaging revealed extensive fluorodeoxyglucose (FDG) uptake in the bilateral lungs, chest lymph nodes, and the upper portion of the right tibia, indicative of a relapse of AITL (**Figure 1B**). He declined an invasive biopsy for pathological evaluation. Following the loss of response to chidamide, the patient refused chemotherapy and chose salvage treatment with lenalidomide at 10 mg daily (for 21 days of a 28-day cycle) in combination with prednisone at 20 mg daily (**Figure 1B**). Prednisone was gradually tapered and discontinued over one month. Subsequently, his ongoing therapy was switched to single-agent lenalidomide at 20 mg daily (for 21 days of a 28-day cycle). Follow-up radiographs revealed a remarkable resolution of all pulmonary lesions after just two cycles of treatment, leading to a fourth CR in July 2016 (**Figure 1B**).

Lenalidomide was administered at the original dose for maintenance therapy with clinical follow-ups every three months. Adverse events included multiple episodes of pneumonia requiring hospitalization, grade 2 thrombocytopenia, and neutropenia. Despite these, the patient returned to a near-normal life. Remarkably, remission persisted for 64 months until recurrence in November 2021. PET/CT imaging revealed multiple lymphadenopathies with increased FDG uptake in the neck, chest, and abdomen. Histology of the left cervical lymph node confirmed relapsed AITL complicated by reactive B-cell proliferation. Notably, the patient had self-administered lenalidomide occasionally for the preceding three months without close monitoring. Upon resuming the prescribed lenalidomide regimen combined with short-term prednisone, all enlarged lymph nodes progressively decreased and mostly disappeared by February 2022, as confirmed by contrast-enhanced CT scans. Nevertheless, due to the refusal of a follow-up PET/CT scan, the response evaluation remained inconclusive, categorized as PR or unconfirmed CR (CRu).

**Figure 2:**
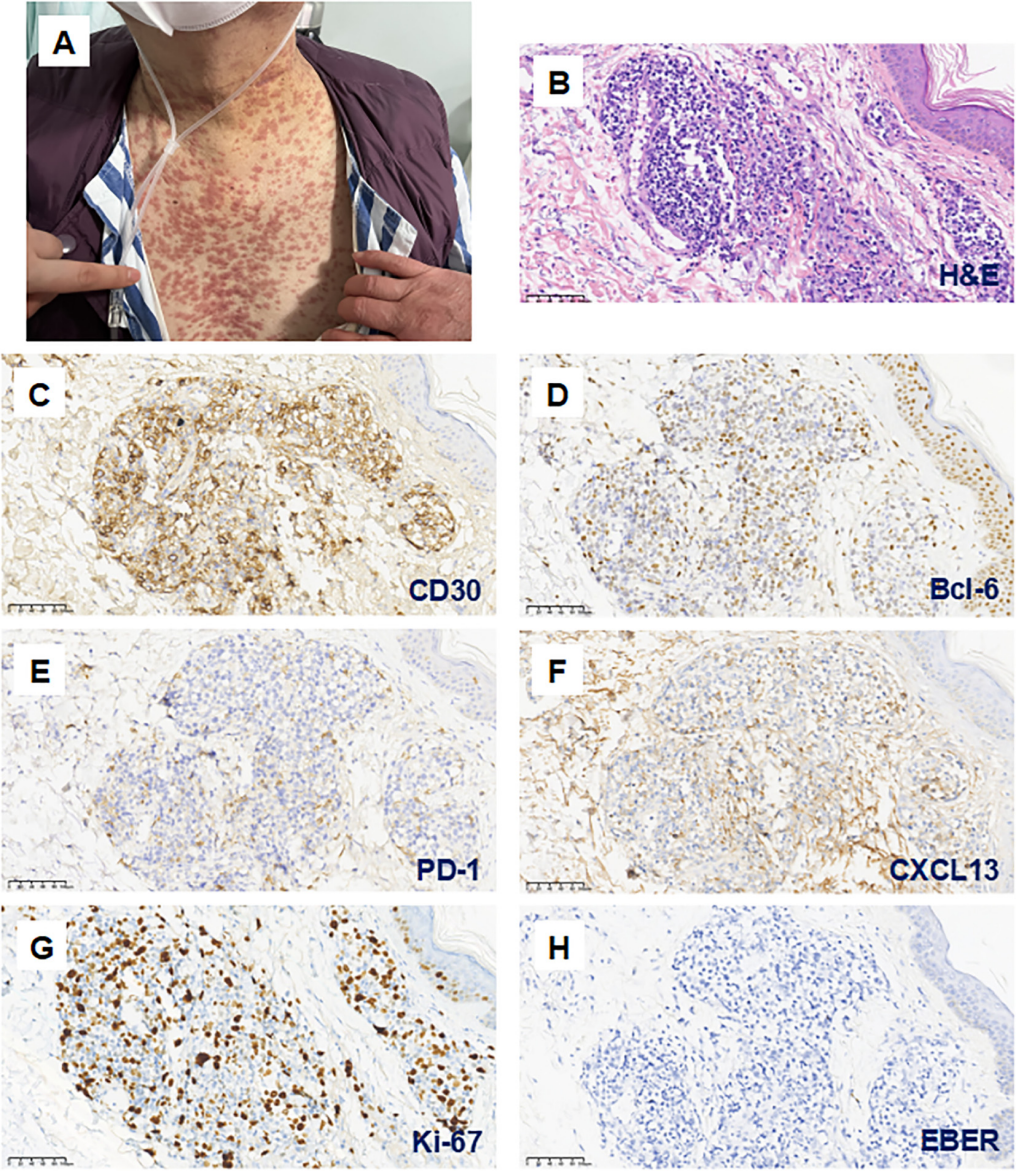
Cutaneous infiltration of AITL concurrent with COVID-19 infection during the fifth relapse. **(A)** After discontinuing Lenalidomide due to severe COVID-19 pneumonia in January 2023, the patient developed widespread pruritic erythematous nodules on the trunk. (**B–H**; magnification 200×) These skin lesions were confirmed as AITL infiltration. Hematoxylin and eosin (H&E) staining demonstrates lymphocytic infiltration surrounding the dermal blood vessels and skin appendages **(B)**. Immunohistochemical analysis showed partial expression of CD30 **(C)** and Bcl-6 **(D)**, with weak positivity for PD-1 **(E)** and CXCL13 **(F)**. The Ki-67 proliferation index was 50% **(G)**, and EBER staining was negative **(H)**.

Despite achieving an additional 11 months of remission with the second round of lenalidomide, the patient’s overall health declined due to recurrent pulmonary infections. He experienced pneumocystis pneumonia in August 2022 and severe COVID-19 pneumonia complicated by Candida glabrata pneumonia in January 2023, which led to the discontinuation of lenalidomide. Subsequently, he developed disseminated pruritic erythematous nodules on his trunk without eosinophilia (**Figure 2A**). Initially, these nodules were attributed to cutaneous manifestations of COVID-19 due to a prompt response to nirmatrelvir/ritonavir (Paxlovid) antiviral treatment. However, a skin biopsy revealed EBER-negative AITL infiltration with a high Ki-67 index of 50% (**Figures 2B–H**). It exhibited partial positivity for Bcl-6, weak positivity for CD10, PD-1, and CXCL13, markers typically associated with TFH cells (**Figures 2D–F**). CD30 expression was observed in 30-40% of tumor cells (**Figure 2C**), along with TCR clonal rearrangement and absence of IgH clonal rearrangement.

Whole-exome sequencing (WES) and low-depth whole-genome sequencing (CNV-seq) were performed to analyze the formalin-fixed, paraffin-embedded (FFPE) skin tissue sample (Kindstar Global Technology, Inc. Wuhan, China). A total of 5 strong clinically significant variants were identified, comprising KMT2D c.5176C>T(p.Gln1726Ter), ARID1A c.4689dup(p.Met1564HisfsTer8), cha(3) (p26.3q28), del(5) (q14.3q23.3), and del(6) (q16.1q22.33) (20, 21) (**Supplementary Tables 1**, **2**). Additionally, FYN c.467G>A (p.Arg156Gln) and dup(13) (q13.1q14.2) were classified as the potentially clinically significant variants (20, 21) (**Supplementary Tables 1**, **2**). No mutations associated with susceptibility to hematological malignancies were detected. Genes frequently mutated in AITL, such as RHOA, TET2, DNMT3A and IDH2 were not identified in our patient, which were also negative in lymph node samples taken at the first relapse by a retrospective targeted sequencing.

As the disease progressed, the patient exhibited progressive diffuse lymphadenopathy (up to 8×9cm in the left preauricular area), splenomegaly, and bone marrow involvement (0.05% of all nucleated cells detected by flow cytometry) but showed no response to retreatment with lenalidomide. Considering the patient’s frailty and the CD30 expression, a single dose of brentuximab vedotin was administered in March 2023, resulting in a transient reduction in the size of the enlarged lymph nodes. Unfortunately, his general condition continued to deteriorate. Laboratory studies indicated persistent pancytopenia, hypoglobulinemia, liver dysfunction, and increased lactate dehydrogenase. He experienced severe pneumonia caused by a range of pathogens, including acinetobacter, candida, H1N1 influenza, and a second wave of COVID-19. Despite exhaustive efforts to combat the aggressive illness, the patient died in June 2023 following a decade-long battle with AITL.

**Figure 3:**
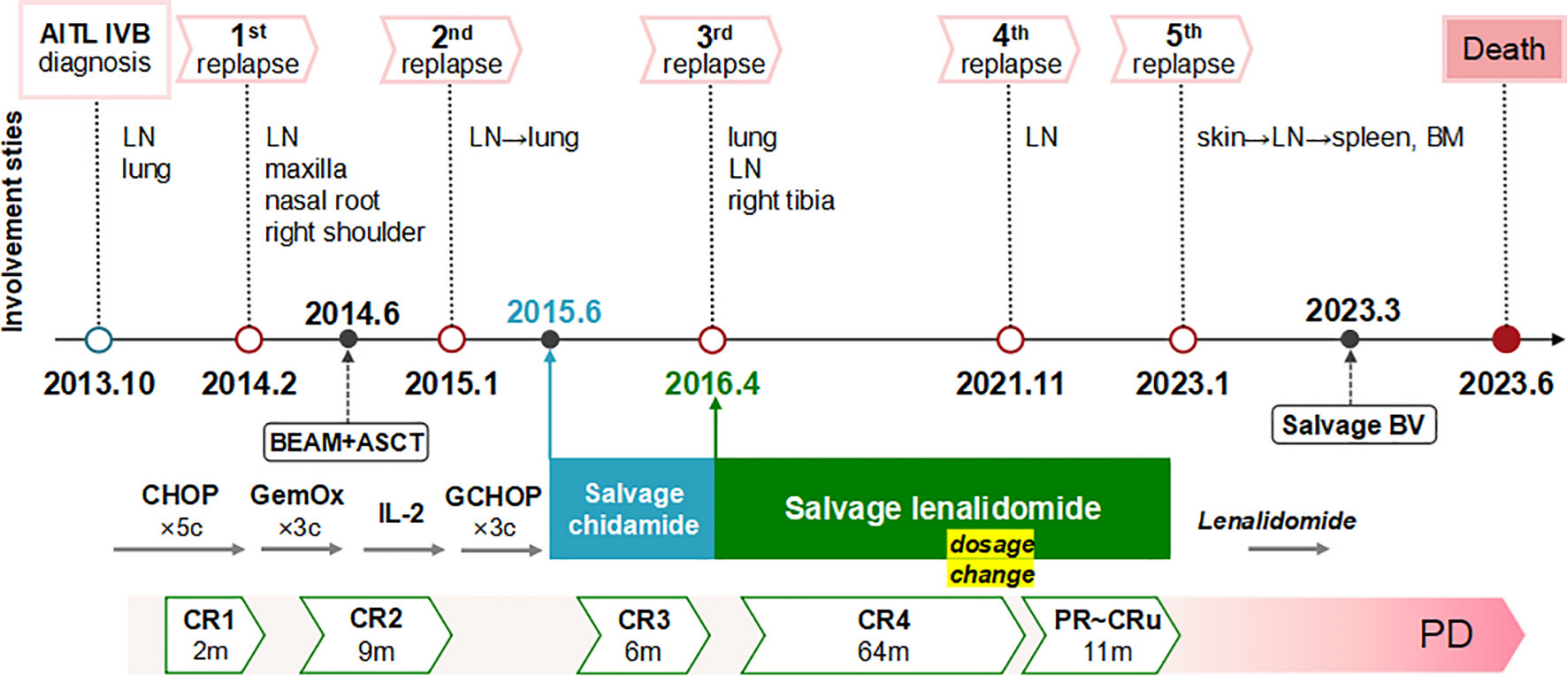
Ten-year clinical course and treatment timeline with relevant data. The patient experienced five relapses over ten years of AITL, with extensive nodal and extranodal involvement. He maintained chemo-free treatments after intensive chemotherapies and ASCT. Good responses were achieved with single-agent chidamide and lenalidomide, notably resolving multiple lung lesions. The response durations were 6 months for chidamide and 75 months for lenalidomide (comprising 64 months of CR and an additional 11 months of diminished response due to self-tapering and subsequent dosage resumption). Following a rapid deterioration after a COVID-19 infection in January 2023, the patient progressed to end-stage disease characterized by disseminated extranodal involvement of the skin, spleen, and bone marrow, loss of response to lenalidomide, limited efficacy of BV salvage therapy, severe immunodeficiency, and recurrent infections, ultimately culminating in his death. AITL, angioimmunoblastic T-cell lymphoma; LN, lymph node; BM, bone marrow; BV, brentuximab vedotin; CR, complete remission; PR, partial remission; CRu, unconfirmed CR; PD, progression of disease.

The ten-year clinical progression and treatment timeline, along with pertinent data were illustrated in **Figure 3**.

## Discussion

Our patient’s decade-long struggle with AITL was marked by frequent relapses and infections, highlighting the formidable challenges in managing relapsed and refractory (R/R) AITL. These challenges encompass selecting appropriate salvage therapies, achieving long-term remission or potential cure, and addressing immune deficiencies arising from the disease or its treatment modalities. These issues become particularly pronounced after the failure of initial intensive chemotherapy and ASCT consolidation, which often lead to patient frailty and indicate a poor prognosis. This case is significant as it exemplifies the use of chemo-free strategies, specifically the successful utilization of chidamide and lenalidomide as salvage therapies. Of particular interest is the remarkable disease control achieved by lenalidomide following the emergence of chidamide resistance. This resulted in over five years of disease-free survival (DFS) with continuous maintenance therapy, despite the eventual decline in response.

As the first oral selective histone deacetylase (HDAC) inhibitor, chidamide was developed in China and approved for the treatment of R/R PTCL in December 2014 (22). Consequently, it emerged as a favored therapeutic option for our patient upon relapse after ASCT in 2015. By enhancing the acetylation of histone and non-histone proteins, chidamide modulates gene expression, induces cell cycle arrest and apoptosis, and inhibits cell growth (23, 24). This unique mechanism holds promise in AITL, a disease characterized by pervasive epigenetic dysregulation (19, 25–29). Clinical studies have shown that chidamide monotherapy is more effective in R/R AITL, achieving a higher overall response rate (ORR) of around 50% and more durable responses compared to other PTCL subtypes (22, 30–32). Regarding our patient, the complete resolution of pulmonary lesions underscores the potential efficacy of single-agent chidamide in managing R/R AITL. Notably, variants with strong clinical significance identified in our patient involved the genes KMT2D and ARID1A. These genes are known to play roles in histone modification and chromatin remodeling, respectively, and are commonly mutated in PTCL, as reported in the genomic and transcriptomic profiling study conducted by Huang et al (33). Given the presence of KMT2D mutation and the lack of common AITL mutations (RHOA, TET2, DNMT3A, IDH2), it is probable that our patient falls within the T3.1 molecular subtype, which is distinguished by mutations related to histone modification and a heightened sensitivity to HDAC inhibitors such as chidamide (33).

However, the 6-month DFS of our patient highlights the challenge of chidamide resistance and the necessity for further research into its underlying mechanisms and alternative treatment strategies. While our understanding of this resistance is limited, research in natural killer/T-cell lymphoma (NKTCL) has suggested a role for aberrant JAK-STAT signaling in primary resistance to chidamide, with TNFRSF8 (CD30) as a potential predictive biomarker (34). Targeting the JAK-STAT signaling may offer a promising strategy to overcome this resistance (34). Nevertheless, the specific mechanisms of chidamide resistance in AITL remain to be elucidated. On the other hand, after enduring numerous chemotherapies and chidamide treatments, the combination of lenalidomide and prednisone as a salvage therapy led to an inspiring fourth CR in our patient. Furthermore, continuous maintenance therapy with lenalidomide alone resulted in a prolonged DFS of 64 months, with manageable hematological and infectious side effects. However, the fourth recurrence of AITL occurred after the patient self-tapered lenalidomide. Even after resuming the original prescription, he experienced no more than 11 months of diminished response. This suggests that lenalidomide monotherapy remained insufficient for achieving sustained deep molecular remission and complete disease eradication in this specific case.

Given the unique biological features of AITL, the immunomodulatory and anti-angiogenic properties of lenalidomide suggest potential therapeutic advantages (35). Lenalidomide demonstrated modest single-agent activity in R/R PTCL, particularly in AITL, with an ORR of 31%~33%, a CR/CRu rate of 11%~15%, and a median progression-free survival (PFS) of 4.6 months (36, 37). Over the past 11 years, sporadic reports have documented the dramatic response to single-agent lenalidomide, with or without corticosteroids or previously used drugs, in patients with R/R AITL, as summarized in **Table 1** (9, 38–42). These cases highlight the considerable potential of lenalidomide, even in heavily pretreated patients. Notably, our case stands out with the longest documented durations of response (DOR) and overall survival (OS), reaching 75 months and 116 months, respectively. Apart from our case, two other patients successfully overcame resistance to HDAC inhibitors with lenalidomide, with one achieving significant improvement in pulmonary lesions (41, 42). Nevertheless, their observed DOR were transient due to either patient death or limited follow-up (41, 42). Importantly, the molecular characteristics of these cases remain unreported, further distinguishing our case. Although a multicenter phase 2 trial found that adding lenalidomide to CHOP did not improve complete metabolic response (CMR) rates compared to historical controls in previously untreated elderly AITL patients (43), lenalidomide-based therapy still shows potential efficacy in treating AITL.

**Table 1:** Case reports of single-agent lenalidomide salvage in relapsed/refractory AITL.

Reference	Case No./Sex/Age(y)	Stage	Prior lines of therapy	Regions of involvement before lenalidomide	Lenalidomide Treatment				OS(mo)	Last follow-up status
					Daily dose	Combination*	Best response	DoR(mo)		
Fabbri et al., 2013 (38)	1/M/59	IVB	3 lines: DHAC, GEMOX, IGEV+radiotherapy	LN, spleen, BM	25mg→15mg	No	CR	42	51	AIR
Beckers et al., 2013 (39)	2/M/54	IVB	2 lines: CHOP, DHAP, palliative prednisone	LN	15mg	PDN	CR	24	32	AIR
Broccoli et al., 2014 (40)	3/F/60	IVB	2 lines: steroid, CHOP, salvage BEAM-ASCT	LN, spleen, BM, hip bones	10mg→15mg	No	CR	19	40	AIR
Kishimoto et al., 2019 (9)	4/F/87	IIIB†	1 lines: R-mini-CHOP, R-CHOP	LN	15mg→20mg	DXM for C1-2	PR	12	~18	Die of liver failure
Sawhney et al., 2020 (41)	5/F/76	IIIB	3 lines: CHOEP, BV, ASCT, romidepsin	LN, pericardium	NA	No	Response#	5	NA	Die of septic shock
Hu et al., 2020 (42)	6/F/62	IVB	4 lines: Chi+CHOP, Chi+DHAP, Chi+MA, ASCT, Chi	lungs	25mg qod	Chi	CRu	6	51	AIR
Our case	7/M/54	IVB	4 lines: CHOP, GEMOX, ASCT, GCHOP, Chi	LN, lungs, tibia	10mg→20mg	PDN for C1	CR	64 + 11 = 75$	116	Die of disease

† AITL complicated by EBV-positive B cell and monoclonal plasma cell proliferation

* Combination drugs specifically referring to corticosteroids or previously used agents.

# patient responded with resolved pericardial effusion, while precise data for response evaluation were unavailable.

$ 64 months of CR, followed by 11 months of diminished response after lenalidomide dosage adjustment.

DHAC, dexamethasone, cytarabine, and pegylated liposomal doxorubicin CaelyxW; GEMOX, gemcitabine and oxaliplatin; IGEV, ifosfamide, gemcitabine, vinorelbine, and prednisone; CHOP, cyclophosphamide, doxorubicin, vincristine, and prednisone; DHAP, dexamethasone, cytarabine, and cisplatin; BEAM, carmustine, etoposide, cytarabine, and melphalan; ASCT, autologous stem cell transplantation; R, rituximab; CHOEP, CHOP and etoposide; BV, brentuximab vedotin; Chi, chidamide; MA, methotrexate and cytarabine; GCHOP, CHOP and gemcitabine; NA, not available; PDN, prednisone; DXM, dexamethasone; C, cycle; CR, complete remission; PR, partial remission; CRu, unconfirmed CR; DoR, duration of response; OS, overall survival; AIR, alive in remission.

Besides direct cytotoxicity and anti-angiogenesis, immunomodulatory drug (IMiD) lenalidomide primarily exerts its activity against hematological malignancies through immunomodulatory mechanisms (35). It impacts cytokine production, enhances T-cell co-stimulation, promotes a Th1 response, boosts NK cell cytotoxicity, and potentiates antibody-dependent cell-mediated cytotoxicity (ADCC) (35). Therefore, the variable efficacy of lenalidomide in AITL individuals may largely be attributed to differences in tumor immune microenvironments and their interactions with malignant TFH cells. Huang et al. categorized four lymphoma microenvironment (LME) subtypes of PTCL through unsupervised clustering of RNA-seq data from immune-related genes: TFH-like, inflammatory, mesenchymal, and depleted (33). Notably, AITL is predominantly associated with the TFH-like or inflammatory subtype (33). In the TFH-like subtype, overexpression of cereblon (CRBN), a crucial binding target of immunomodulatory agents, suggests a potential favorable response to lenalidomide (33). However, despite our case showing marked sensitivity to lenalidomide, its mutational profile did not align with the defined TFH-like subtype, which is characterized by TFH cell origin, enriched TFH markers, high B/plasma cell content, and molecular associations with TET2 and RHOA mutations (33). Without RNA-seq analysis before lenalidomide administration, his LME subtype cannot be precisely determined. This emphasizes the complexity of the AITL microenvironment and the difficulties in selecting targeted therapy.

The mechanism underlying acquired resistance to lenalidomide in AITL remains poorly understood, with most data focused on multiple myeloma. Potential factors include mutations or reduced expression of CRBN, altered protein degradation pathways or signaling pathways, and remodeling of the immune microenvironment (44–50). Considering the decreasing efficacy of lenalidomide in the later stages of the disease and the heightened vulnerability to infections, immune exhaustion may play a contributory role in our case.

In addition, studies on the cytogenomics of AITL remain limited. Nelson et al. identified common genetic imbalances among 22 cases, including gains in 5q, 21, and 3q, concurrent trisomies of 5 and 21, and loss of 6q (51). Our case presented multiple clinically significant chromosomal aberrations, including the previously mentioned del 6q. Of particular interest is the detection of del(5)(q14.3q23.3), typically associated with del 5q syndrome, a subtype of myelodysplastic syndrome (MDS) known for its responsiveness to lenalidomide (52, 53). However, the implications and relationship of these findings with lenalidomide sensitivity in AITL require further investigation.

## Conclusion

Despite the emergence of numerous targeted therapies and immunotherapies, lenalidomide has proven to be an effective, tolerable, affordable, and convenient option for AITL, particularly by targeting its immune dysregulation and hypervascularity. Our case highlights the enduring effectiveness of lenalidomide in treating serious R/R AITL, including patients with extensive prior treatments, resistance to HDAC inhibitors, and widespread extranodal involvement, notably in the lungs. Continuation of lenalidomide maintenance is advisable for patients experiencing recurrent relapses or failing to achieve deep remission, and exploration of non-invasive monitoring of minimal residual disease (MRD) and immune dynamics in these patients may be imperative. With our enhanced understanding of the molecular pathogenesis and resistance mechanisms of AITL, encompassing genetic, epigenetic, and transcriptional processes in both malignant cells and their microenvironment, the precise utilization of chemo-free treatments guided by biomarker-driven approaches will be further optimized.

## Data Availability

The original contributions presented in the study are included in the article/**Supplementary Materials**. Further inquiries can be directed to the corresponding authors.

## References

[1] Alaggio R Amador C Anagnostopoulos I Attygalle AD Araujo IBO Berti E . The 5th edition of the world health organization classification of haematolymphoid tumours: lymphoid neoplasms. Leukemia. (2022) 36:1720–48. doi: 10.1038/s41375-022-01620-2 PMC9214472 35732829

[2] Federico M Rudiger T Bellei M Nathwani BN Luminari S Coiffier B . Clinicopathologic characteristics of angioimmunoblastic T-cell lymphoma: analysis of the international peripheral T-cell lymphoma project. J Clin oncology: Off J Am Soc Clin Oncol. (2013) 31:240–6. doi: 10.1200/JCO.2011.37.3647 PMC3532394 22869878

[3] Lunning MA Vose JM . Angioimmunoblastic T-cell lymphoma: the many-faced lymphoma. Blood. (2017) 129:1095–102. doi: 10.1182/blood-2016-09-692541 28115369

[4] Gaulard P de Leval L . The microenvironment in T-cell lymphomas: emerging themes. Semin Cancer Biol. (2014) 24:49–60. doi: 10.1016/j.semcancer.2013.11.004 24316493

[5] Chiba S Sakata-Yanagimoto M . Advances in understanding of angioimmunoblastic T-cell lymphoma. Leukemia. (2020) 34:2592–606. doi: 10.1038/s41375-020-0990-y PMC7376827 32704161

[6] Huppmann AR Roullet MR Raffeld M Jaffe ES . Angioimmunoblastic T-cell lymphoma partially obscured by an Epstein-Barr virus-negative clonal plasma cell proliferation. J Clin oncology: Off J Am Soc Clin Oncol. (2013) 31:e28–30. doi: 10.1200/JCO.2012.43.3797 PMC3532397 23213091

[7] Xu J Tang Y Zhao S Zhang W Xiu Y Liu T . Angioimmunoblastic T-cell lymphoma with coexisting plasma cell myeloma: a case report and review of the literature. Tohoku J Exp Med. (2015) 235:283–8. doi: 10.1620/tjem.235.283 25816919

[8] Okuyama S Terada T Kumagai H Tsumanuma R Omoto E Ueki T . Epstein-Barr virus clonality and plasmacytosis in a patient with atypical angioimmunoblastic T cell lymphoma. Ann hematology. (2018) 97:537–9. doi: 10.1007/s00277-017-3189-1 29189897

[9] Kishimoto W Takiuchi Y Nakae Y Tabata S Fukunaga A Matsuzaki N . A case of AITL complicated by EBV-positive B cell and monoclonal plasma cell proliferation and effectively treated with lenalidomide. Int J hematology. (2019) 109:499–504. doi: 10.1007/s12185-018-02587-6 30604313

[10] Mejia Saldarriaga M Alhomoud M Roboz G Allan JN Ruan J Ouseph MM . Angioimmunoblastic T-cell lymphoma presenting with severe plasmacytosis mimicking plasma cell leukemia. Am J hematology. (2023) 98:1119–26. doi: 10.1002/ajh.26878 36785525

[11] Lage L Culler HF Reichert CO da Siqueira SAC Pereira J . Angioimmunoblastic T-cell lymphoma and correlated neoplasms with T-cell follicular helper phenotype: from molecular mechanisms to therapeutic advances. Front Oncol. (2023) 13:1177590. doi: 10.3389/fonc.2023.1177590 37182145 PMC10169672

[12] Vallois D Dobay MP Morin RD Lemonnier F Missiaglia E Juilland M . Activating mutations in genes related to TCR signaling in angioimmunoblastic and other follicular helper T-cell-derived lymphomas. Blood. (2016) 128:1490–502. doi: 10.1182/blood-2016-02-698977 27369867

[13] Mourad N Mounier N Briere J Raffoux E Delmer A Feller A . Clinical, biologic, and pathologic features in 157 patients with angioimmunoblastic T-cell lymphoma treated within the Groupe d'Etude des Lymphomes de l'Adulte (GELA) trials. Blood. (2008) 111:4463–70. doi: 10.1182/blood-2007-08-105759 PMC2343588 18292286

[14] Vose J Armitage J Weisenburger D International TCLP . International peripheral T-cell and natural killer/T-cell lymphoma study: pathology findings and clinical outcomes. J Clin oncology: Off J Am Soc Clin Oncol. (2008) 26:4124–30. doi: 10.1200/JCO.2008.16.4558 18626005

[15] Broccoli A Zinzani PL . Angioimmunoblastic T-cell lymphoma. Hematology/oncology Clinics North America. (2017) 31:223–38. doi: 10.1016/j.hoc.2016.12.001 28340875

[16] Advani RH Skrypets T Civallero M Spinner MA Manni M Kim WS . Outcomes and prognostic factors in angioimmunoblastic T-cell lymphoma: final report from the international T-cell Project. Blood. (2021) 138:213–20. doi: 10.1182/blood.2020010387 PMC8493974 34292324

[17] Wei C Li W Qin L Liu S Xue C Ren K . Clinicopathologic characteristics, outcomes, and prognostic factors of angioimmunoblastic T-cell lymphoma in China. Cancer Med. (2023) 12:3987–98. doi: 10.1002/cam4.v12.4 PMC9972121 36106610

[18] Mak V Hamm J Chhanabhai M Shenkier T Klasa R Sehn LH . Survival of patients with peripheral T-cell lymphoma after first relapse or progression: spectrum of disease and rare long-term survivors. J Clin oncology: Off J Am Soc Clin Oncol. (2013) 31:1970–6. doi: 10.1200/JCO.2012.44.7524 23610113

[19] Zhang Q Yin L Lai Q Zhao Y Peng H . Advances in the pathogenesis and therapeutic strategies of angioimmunoblastic T-cell lymphoma. Clin Exp Med. (2023) 23:4219–35. doi: 10.1007/s10238-023-01197-9 37759042

[20] Li MM Datto M Duncavage EJ Kulkarni S Lindeman NI Roy S . Standards and guidelines for the interpretation and reporting of sequence variants in cancer: A joint consensus recommendation of the association for molecular pathology, American society of clinical oncology, and college of American pathologists. J Mol Diagn. (2017) 19:4–23. doi: 10.1016/j.jmoldx.2016.10.002 27993330 PMC5707196

[21] Mikhail FM Biegel JA Cooley LD Dubuc AM Hirsch B Horner VL . Technical laboratory standards for interpretation and reporting of acquired copy-number abnormalities and copy-neutral loss of heterozygosity in neoplastic disorders: a joint consensus recommendation from the American College of Medical Genetics and Genomics (ACMG) and the Cancer Genomics Consortium (CGC). Genet Med. (2019) 21:1903–16. doi: 10.1038/s41436-019-0545-7 31138931

[22] Shi Y Dong M Hong X Zhang W Feng J Zhu J . Results from a multicenter, open-label, pivotal phase II study of chidamide in relapsed or refractory peripheral T-cell lymphoma. Ann oncology: Off J Eur Soc Med Oncol / ESMO. (2015) 26:1766–71. doi: 10.1093/annonc/mdv237 26105599

[23] Ning ZQ Li ZB Newman MJ Shan S Wang XH Pan DS . Chidamide (CS055/HBI-8000): a new histone deacetylase inhibitor of the benzamide class with antitumor activity and the ability to enhance immune cell-mediated tumor cell cytotoxicity. Cancer chemotherapy Pharmacol. (2012) 69:901–9. doi: 10.1007/s00280-011-1766-x 22080169

[24] Cao HY Li L Xue SL Dai HP . Chidamide: Targeting epigenetic regulation in the treatment of hematological Malignancy. Hematological Oncol. (2023) 41:301–9. doi: 10.1002/hon.v41.3 36251458

[25] Cairns RA Iqbal J Lemonnier F Kucuk C de Leval L Jais JP . IDH2 mutations are frequent in angioimmunoblastic T-cell lymphoma. Blood. (2012) 119:1901–3. doi: 10.1182/blood-2011-11-391748 PMC3293643 22215888

[26] Palomero T Couronne L Khiabanian H Kim MY Ambesi-Impiombato A Perez-Garcia A . Recurrent mutations in epigenetic regulators, RHOA and FYN kinase in peripheral T cell lymphomas. Nat Genet. (2014) 46:166–70. doi: 10.1038/ng.2873 PMC3963408 24413734

[27] Odejide O Weigert O Lane AA Toscano D Lunning MA Kopp N . A targeted mutational landscape of angioimmunoblastic T-cell lymphoma. Blood. (2014) 123:1293–6. doi: 10.1182/blood-2013-10-531509 PMC4260974 24345752

[28] Xie C Li X Zeng H Qian W . Molecular insights into pathogenesis and targeted therapy of peripheral T cell lymphoma. Exp Hematol Oncol. (2020) 9:30. doi: 10.1186/s40164-020-00188-w 33292562 PMC7664070

[29] Yoon SE Cho J Kim YJ Ko YH Park WY Kim SJ . Comprehensive analysis of clinical, pathological, and genomic characteristics of follicular helper T-cell derived lymphomas. Exp Hematol Oncol. (2021) 10:33. doi: 10.1186/s40164-021-00224-3 33990228 PMC8120779

[30] Shi Y Jia B Xu W Li W Liu T Liu P . Chidamide in relapsed or refractory peripheral T cell lymphoma: a multicenter real-world study in China. J Hematol Oncol. (2017) 10:69. doi: 10.1186/s13045-017-0439-6 28298231 PMC5351273

[31] Chan TS Tse E Kwong YL . Chidamide in the treatment of peripheral T-cell lymphoma. OncoTargets Ther. (2017) 10:347–52. doi: 10.2147/OTT.S93528 PMC5238768 28138258

[32] Yang P Tao Y Zhao A Shen K Li H Wang J . Efficacy and safety of histone deacetylase inhibitors in peripheral T-cell lymphoma: a systematic review and meta-analysis on prospective clinical trials. Front Oncol. (2023) 13:1127112. doi: 10.3389/fonc.2023.1127112 37384289 PMC10293743

[33] Huang YH Qiu YR Zhang QL Cai MC Yu H Zhang JM . Genomic and transcriptomic profiling of peripheral T cell lymphoma reveals distinct molecular and microenvironment subtypes. Cell Rep Med. (2024) 5:101416. doi: 10.1016/j.xcrm.2024.101416 38350451 PMC10897627

[34] Chen J Zuo Z Gao Y Yao X Guan P Wang Y . Aberrant JAK-STAT signaling-mediated chromatin remodeling impairs the sensitivity of NK/T-cell lymphoma to chidamide. Clin Epigenetics. (2023) 15:19. doi: 10.1186/s13148-023-01436-6 36740715 PMC9900953

[35] Kotla V Goel S Nischal S Heuck C Vivek K Das B . Mechanism of action of lenalidomide in hematological Malignancies. J Hematol Oncol. (2009) 2:36. doi: 10.1186/1756-8722-2-36 19674465 PMC2736171

[36] Morschhauser F Fitoussi O Haioun C Thieblemont C Quach H Delarue R . A phase 2, multicentre, single-arm, open-label study to evaluate the safety and efficacy of single-agent lenalidomide (Revlimid) in subjects with relapsed or refractory peripheral T-cell non-Hodgkin lymphoma: the EXPECT trial. Eur J Cancer. (2013) 49:2869–76. doi: 10.1016/j.ejca.2013.04.029 23731832

[37] Toumishey E Prasad A Dueck G Chua N Finch D Johnston J . Final report of a phase 2 clinical trial of lenalidomide monotherapy for patients with T-cell lymphoma. Cancer. (2015) 121:716–23. doi: 10.1002/cncr.v121.5 25355245

[38] Fabbri A Cencini E Pietrini A Gozzetti A Defina M Fontanelli G . Impressive activity of lenalidomide monotherapy in refractory angioimmunoblastic T-cell lymphoma: report of a case with long-term follow-up. Hematological Oncol. (2013) 31:213–7. doi: 10.1002/hon.v31.4 23161606

[39] Beckers MM Huls G . Therapy refractory angioimmunoblastic T-cell lymphoma in complete remission with lenalidomide. Eur J Haematol. (2013) 90:162–3. doi: 10.1111/ejh.2013.90.issue-2 23227803

[40] Broccoli A Pellegrini C Celli M Argnani L Agostinelli C Pileri S . Single-agent lenalidomide is effective in the treatment of a heavily pretreated and refractory angioimmunoblastic T-cell lymphoma patient. Clin lymphoma myeloma leukemia. (2014) 14:e119–22. doi: 10.1016/j.clml.2014.01.011 24629851

[41] Sawhney R Volkmer RD 2nd Cooper B . Relapsed angioimmunoblastic T-cell lymphoma with large pericardial effusion. Proc (Bayl Univ Med Cent). (2020) 33:62–4. doi: 10.1080/08998280.2019.1668720 PMC6988641 32063773

[42] Hu S Zou D Zhou D Zhang Y Wang W Zhang W . Successful treatment with lenalidomide plus chidamide combination therapy in 3 heavily treated patients with non-Hodgkin lymphoma: Three cases report. Medicine. (2020) 99:e22788. doi: 10.1097/MD.0000000000022788 33120793 PMC7581128

[43] Lemonnier F Safar V Beldi-Ferchiou A Cottereau AS Bachy E Cartron G . Integrative analysis of a phase 2 trial combining lenalidomide with CHOP in angioimmunoblastic T-cell lymphoma. Blood Adv. (2021) 5:539–48. doi: 10.1182/bloodadvances.2020003081 PMC7839364 33496747

[44] Franssen LE Nijhof IS Couto S Levin MD Bos GMJ Broijl A . Cereblon loss and up-regulation of c-Myc are associated with lenalidomide resistance in multiple myeloma patients. Haematologica. (2018) 103:e368–e71. doi: 10.3324/haematol.2017.186601 PMC6068039 29545338

[45] Gooding S Ansari-Pour N Towfic F Ortiz Estévez M Chamberlain PP Tsai KT . Multiple cereblon genetic changes are associated with acquired resistance to lenalidomide or pomalidomide in multiple myeloma. Blood. (2021) 137:232–7. doi: 10.1182/blood.2020007081 PMC7893409 33443552

[46] Haertle L Barrio S Munawar U Han S Zhou X Vogt C . Cereblon enhancer methylation and IMiD resistance in multiple myeloma. Blood. (2021) 138:1721–6. doi: 10.1182/blood.2020010452 PMC8569411 34115836

[47] Bjorklund CC Ma W Wang ZQ Davis RE Kuhn DJ Kornblau SM . Evidence of a role for activation of Wnt/beta-catenin signaling in the resistance of plasma cells to lenalidomide. J Biol Chem. (2011) 286:11009–20. doi: 10.1074/jbc.M110.180208 PMC3064156 21189262

[48] Bjorklund CC Baladandayuthapani V Lin HY Jones RJ Kuiatse I Wang H . Evidence of a role for CD44 and cell adhesion in mediating resistance to lenalidomide in multiple myeloma: therapeutic implications. Leukemia. (2014) 28:373–83. doi: 10.1038/leu.2013.174 PMC3874423 23760401

[49] Zhu YX Shi CX Bruins LA Wang X Riggs DL Porter B . Identification of lenalidomide resistance pathways in myeloma and targeted resensitization using cereblon replacement, inhibition of STAT3 or targeting of IRF4. Blood Cancer J. (2019) 9:19. doi: 10.1038/s41408-019-0173-0 30741931 PMC6370766

[50] Lucas F Pennell M Huang Y Benson DM Efebera YA Chaudhry M . T cell transcriptional profiling and immunophenotyping uncover LAG3 as a potential significant target of immune modulation in multiple myeloma. Biol Blood marrow transplantation: J Am Soc Blood Marrow Transplantation. (2020) 26:7–15. doi: 10.1016/j.bbmt.2019.08.009 PMC6952061 31445183

[51] Nelson M Horsman DE Weisenburger DD Gascoyne RD Dave BJ Loberiza FR . Cytogenetic abnormalities and clinical correlations in peripheral T-cell lymphoma. Br J haematology. (2008) 141:461–9. doi: 10.1111/j.1365-2141.2008.07042.x 18341637

[52] Nimer SD . Clinical management of myelodysplastic syndromes with interstitial deletion of chromosome 5q. J Clin oncology: Off J Am Soc Clin Oncol. (2006) 24:2576–82. doi: 10.1200/JCO.2005.03.6715 16735711

[53] Fink EC Ebert BL . The novel mechanism of lenalidomide activity. Blood. (2015) 126:2366–9. doi: 10.1182/blood-2015-07-567958 PMC4653765 26438514

