# Supplementary material for: Sustained yet non-curative response to lenalidomide in relapsed angioimmunoblastic T-cell lymphoma with acquired chidamide resistance: a case report with 10-year follow-up, genetic insights and literature review
Source: Front Oncol. 2024 Nov 27;14:1471090. doi: 10.3389/fonc.2024.1471090 (PMC11631872; doi:10.3389/fonc.2024.1471090)
Supplement: Supplementary file 1 [file DataSheet1.pdf]

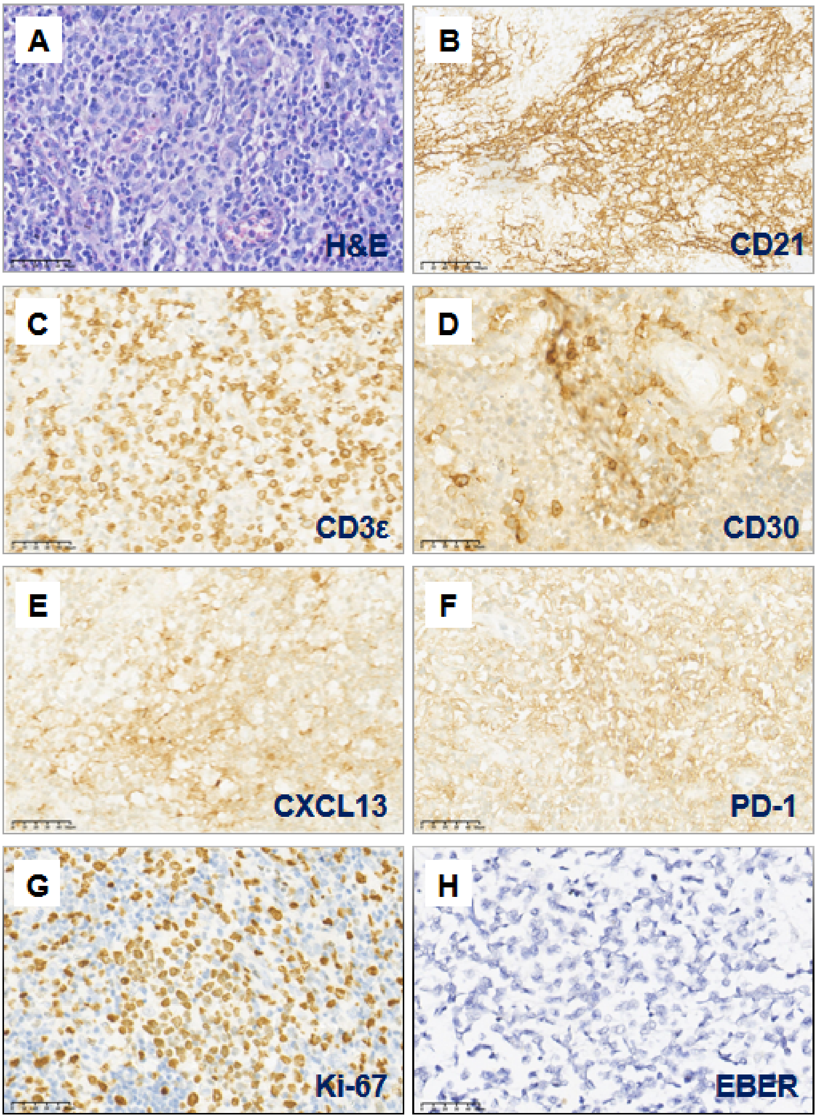

Supplementary Figure 1. Biopsy of cervical lymph node shows characteristic pathological features of AITL.

H&E staining revealed typical pattern III morphology, characterized by follicle effacement, prominent arborizing HEV, polymorphic cellular infiltration, and atypical lymphocytes with clear cytoplasm (**A**; magnification 400×). CD21 immunostaining highlighted an expanded FDC meshwork (**B**, ×200). The neoplastic clear cells were positive for CD3ε (**C**, ×200) and partially positive for CD30, CXCL13, and PD-1 (**D-F**, ×200). The Ki-67 index was 60% (**G**, ×200), and EBER staining was negative (**H**, ×200).
